# P-1159. *S. aureus* α-toxin Neutralization Antibody Reduces Risk for Recurrent Skin and Soft Tissue Infection in Children

**DOI:** 10.1093/ofid/ofae631.1345

**Published:** 2025-01-29

**Authors:** Carol Kao, Jaclyn Wright, Mary G Boyle, Andrea Forbes, Kristina G Hulten, Jonathon C McNeil, Sheldon L Kaplan, Stephanie A Fritz, Juliane Bubeck-Wardenburg

**Affiliations:** Washington University in St Louis School of Medicine, St Louis, Missouri; Washington University School of Medicine, St. Louis, Missouri; Washington University School of Medicine, St. Louis, Missouri; Texas Children's Hospital, Houston, Texas; Baylor College of Medicine, Houston, Texas; Baylor College of Medicine, Houston, Texas; Baylor College of Medicine, Houston, TX; Washington University School of Medicine, St. Louis, Missouri; Washington University School of Medicine in St. Louis

## Abstract

**Background:**

*Staphylococcus aureus* is the leading bacterial cause of infectious-related mortality worldwide. To date, immune correlates of protection are undefined, resulting in unsuccessful vaccine development attempts. Anti-*S. aureus* α-toxin (Hla) IgG response is the only identified correlate of protection against recurrent infection in children. We aimed to correlate the anti-Hla neutralization antibody (Nab) response against risk of recurrent *S. aureus* infection.

**Figure:**
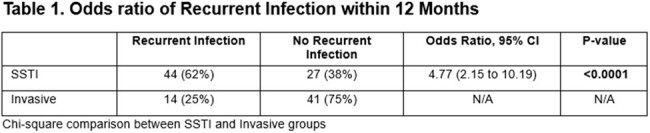

**Methods:**

We utilized serum and plasma samples from two prospective, observational studies enrolling children: 1) healthy controls with or without *S. aureus* colonization, 2) culture-confirmed *S. aureus* skin and soft tissue infection (SSTI), or 3) acute invasive *S. aureus* infection. Patients with risk factors for healthcare-associated *S. aureus* infections were excluded. Acute blood samples were drawn at enrollment/acute infection and convalescent samples 4-8 weeks later. Fold induction titer was calculated as a ratio between the convalescent and acute anti-Hla Nab titer. All participants were followed longitudinally every 3 months for 1 year to document recurrent infection. Anti-Hla IgG was determined by ELISA and anti-Hla Nab titer by RBC lysis assay.

**Results:**

A total of 254 participants were included in the overall analyses. Children with SSTI had a 4.77 (95% CI: 2.15 to 10.19) increased odds of having a recurrent infection and were younger (mean age: 2.51 vs 5.17 years, p< 0.0001) compared to those with invasive infection (**Table 1**). Acute and convalescent titers were compared across patients: children with invasive infections had the highest acute anti-Hla NAb titers while non-colonized controls had the lowest (1.62 vs. 0.55, p< 0.0001). Children with SSTI and no recurrent infections had higher convalescent to acute anti-Hla NAb titers. Among patients with SSTI, for each unit increase in the log fold induction anti-Hla NAb titer, children with SSTI had a 0.54 (95% CI: 0.31-0.96) decreased odds of recurrent infection within 12 months of follow-up. We did not see a similar trend in the invasive group.

**Conclusion:**

Higher anti-Hla NAb fold induction titer correlated significantly with a decreased risk of recurrent SSTI infection. Our study supports the advancement of Hla as an antigenic target for *S. aureus* vaccine development.

**Disclosures:**

**Kristina G. Hulten, PhD**, Pfizer: Advisor/Consultant|Pfizer: Grant/Research Support **Jonathon C. McNeil, MD**, Nabriva: site investigator on clinical trial **Sheldon L. Kaplan, MD**, Pfizer: Grant/Research Support **Juliane Bubeck-Wardenburg, MD, PhD**, Aridis Pharmaceuticals: I have a financial agreement with Aridis Pharmaceuticals related to patents owned by the University of Chicago|Forward Defense, LLC: I may receive royalty income based on a technology developed in my laboratory that is currently owned by Washington University and subject to licens|Forward Defense, LLC: Ownership Interest

